# The Effect of Canal Dryness on Bond Strength of Bioceramic and Epoxy-resin Sealers after Irrigation with Sodium Hypochlorite or Chlorhexidine

**DOI:** 10.7508/iej.2016.02.011

**Published:** 2016-03-20

**Authors:** Hasan Razmi, Behnam Bolhari, Negar Karamzadeh Dashti, Mahta Fazlyab

**Affiliations:** a * Department of Endodontics, Dental School, Dental Research Center, Tehran University of Medical Sciences, Tehran, Iran; *; b * Endodontist, Bushehr University of Medical Sciences, Bushehr, Iran; *; c *Iranian Center for Endodontic Research, Research Institute of Dental Sciences, Dental School, Shahid Beheshti University of Medical Sciences, Tehran, Iran and Department of Endodontics, Dental Branch, Islamic Azad University, Tehran, Iran *

**Keywords:** Bioceramic, Chlorhexidine, Epoxy Resin-Based Root Canal Sealer, Root Canal Sealer, Sodium Hypochlorite

## Abstract

**Introduction:**

The aim of this *in vitro* study was to evaluate the effect of canal dryness on the push-out bond strength of two resin sealers (AH-Plus and Adseal) and a bioceramic sealer (Endosequence BC sealer) after canal irrigation with sodium hypochlorite (NaOCl) and chlorhexidine (CHX).

**Methods and Materials:**

A total of 18 extracted human premolars were used. Canals were prepared and were divided to two groups based on irrigation solution (either NaOCl or CHX). The samples were again divided based on pre-obturation canal condition (wet, half-wet and dry). The samples were sub-divided into 3 groups based on the sealer type; the teeth were obturated with gutta-percha and test sealers (Adseal, AH-Plus or BC sealer). A total number of 18 groups were available to be cut into dentine disks (12 disks in each group). The type of bond failure was also assessed in each group. Data were analyzed using the 3-way ANOVA, post hoc Tukey’s tests, t-test and the Fisher’s exact test. The level of significance was set at 0.05.

**Results:**

The bond strength of Adseal was not affected by the canal condition or irrigation with either NaOCl or CHX. Although the bond strength of AH-Plus was not affected by the irrigant type, the highest bond strength was seen in dry canals. For Endosequence BC sealer, the canal conditions did not affect the bond strength; however, CHX reduced the bond strength.

**Conclusion:**

Bond strength of resin sealers was not affected by irrigation solution; however, canal moisture negatively affected the bond strength of AH-Plus. CHX reduced the bond strength of BC sealer.

## Introduction

Microorganisms and their byproducts are the main etiologic factors of pulpal and periapical diseases [1]. Successful root canal treatment depends on complete debridement, elimination of pathologic organisms and finally complete seal of the root canal system to prevent penetration or re-entry of oral microorganisms into periradicular spaces [2]. Mechanical instrumentation, irrigation with proper disinfecting solutions and use of intracanal medicaments significantly reduce the microbial counts within the infected canals [1]. Although mechanical instrumentation is essential for elimination of root canal infection, it might have limited ability in some parts of the root canal system because of the complex morphology, lateral and accessory canals and apical deltas. Therefore, the root canal system should also be cleaned by means of chemical agents [3].

Irrigants are used not only for antimicrobial purposes but they are also used for lubricating the canal walls, flushing out debris, dissolving organic and inorganic constituents of the smear layer, cleaning dentin surfaces and sometimes improving the capability of resin-based sealers [4]. Irrigation of dentin surface might alter its chemical structure. The changes include a decrease in dentin resistance to stress, predisposing the surface to bacterial adhesion and influencing the bonding of obturation materials to the canal walls [5].

Sodium hypochlorite (NaOCl) is the most common endodontic irrigant. It can dissolve organic tissues, removes organic parts of the smear layer and it is a very potent antimicrobial agent [6]. Chlorhexidine (CHX) is another common endodontic irrigant. CHX is advocated due to its substantivity and antimicrobial effect through binding to hydroxyapatite [3] and yielding antimicrobial activity similar to NaOCl [7]. However, it lacks tissue solubility which is considered a weak point [8].

Although CHX is recommended as an endodontic irrigant, its effect on radicular dentin and bond strength of resin-based sealers is not clear [7]. On the other hand, although NaOCl is an ideal irrigant in many aspects, during its use with adhesive resins it can cause problems because it leaves behind an oxygen-rich layer due to oxidizing activities. This layer results in a decrease in bond strength and an increase in microleakage [9].

Use of gutta-percha with a sealer is the most accepted method of root canal obturation [10]. Sealer is used to fill the irregularities of root canal walls, apical ramifications and deltas [11] along with areas that the main filling material cannot reach [5].

Epoxy resin-based resins have good physical properties, apical sealing ability, adequate biologic function and micro-retention to root dentin [12]. Sealers like AH-plus (Dentsply Maillefer, Tulsa, OK, USA) are frequently used as control materials in endodontic research due to their low solubility, long-lasting dimensional stability and adequate micro-retention to dentin [13]. Adseal (Meta Biomed Co, Cheongju, Korea) is another epoxy resin-based sealer. The manufacturer claims excellent biocompatibility, good sealing and good radiopacity. This sealer is easily mixed. It does not cause tooth discoloration and does not dissolve in tissue fluids [14]. 

Bioceramics consist of nanosphere particles, with the maximum dimension not exceeding 1.9×10^-3^ µm mainly composed of tricalcium silicate, dicalcium silicate, calcium phosphate monobasic, amorphous silicon dioxide and tantalum pentoxide [15]. Due to their ability to penetrate into the dentinal tubules and interact with dentine moisture, an optimum dimensional stability and the least amount of shrinkage can be expected with them. Endosequence BC sealer (Brasseler USA, Savannah, GA, USA) is a new bioceramic sealer with calcium silicate-based composition which is insoluble, radiopaque and aluminum-free [10]. This sealer uses the natural moisture in the dentinal tubules to initiate the setting reaction. This sealer in composed of calcium silicate, monobasic calcium phosphate, calcium hydroxide and zirconium oxide, similar to white MTA [16].

The aim of this study was to compare the bond strength of these three aforementioned sealers, under three different moisture conditions and with the use of two different irrigants. The null hypothesis stated that there is no difference in bond strength of these epoxy resin and bioceramic sealers in different moisture conditions.

## Materials and Methods

In this study, a total number of 216 dentin disks each including a prepared and obturated canal were prepared from 18 single-rooted extracted human premolars with straight roots. 

The sample preparation procedure was as follows: The coronal part of each tooth was removed by using a disk (Brassler, Savannah, GA, USA) to leave almost 16 mm of the root length with no caries. The working length was confirmed by using a #10 K-file (Dentsply Maillefer, Ballaigues, Switzerland) by reducing 1 mm from the file length after its emergence through the apical foramen. Preparation of the root canals was done with F3 ProTaper rotary instruments (Dentsply Maillefer, Tulsa, OK, USA). Irrigation was carried out with saline solution. The smear layer was removed with 10 mL of 17% EDTA for 1 min, followed by irrigation with 10 mL of 5.25% NaOCl, according to the method introduced by Goldman *et al.* [17].

Then the root canals were irrigated with 20 mL of saline solution and were dried with paper points. The teeth were divided into two groups based on the final irrigation: in CHX and NaOCl groups, 2% CHX (Consepsis V, Ultradent Products, Inc., South Jordan, UT, USA) and 5.25% NaOCl were used for irrigation, respectively. The teeth were then divided into 3 groups according to moisture conditions before obturation: *Wet*; the teeth were not dried before obturation, *half-wet*; A #35 paper point was used for 5 sec to partially dry the canal before obturation and *dry; *the root canals were dried by means of paper points until the last paper point showed no moisture and came out totally dry.

Then the teeth were again sub-divided into 3 more groups according to the sealer used beside gutta-percha for obturation: AH-Plus (Dentsply Maillefer, Tulsa, OK, USA), Adseal (Meta Biomed Co, Cheongju, Korea) and Endosequence (Brasseler USA, Savannah, GA, USA). Obturation was carried out in all the groups using the lateral condensation technique. After obturation, the teeth were kept in an incubator at 37^°^C temperature and 90% humidity for one week. The teeth were sectioned into 2-mm-thick disks perpendicular to the long axis of the teeth by means of a cutting machine (Mecatome P100, Presi, France). The disks were prepared from mid-root area and were then observed for circular shape of the core filling material and those not meeting this criterion were eliminated. In each group, 12 disks were collected (*n*=12).

The push-out test was carried out by means of a universal testing machine (Z250, Zwick/Roel, Ulm, Germany) at a crosshead speed of 1 mm/min. The maximum load applied to the core material before the occurrence of bond failure, was recorded in Newton (N). To calculate the bond strength in MPa the bonding surface area was measured using the 2*πrh* formula, where *r* is the diameter of the core material measured under a stereomicroscope and *h* is the height of the disks. The push-out test values in N were divided and the push-out test results were reported in MPa.

The failure modes were determined under a stereomicroscope as follows: *Adhesive failure*, failure at restorative material-dentin interface, *cohesive failure*; failure within the filling material and mixed; a combination of both types. The obtained data were analyzed using the 3-way ANOVA, post hoc tests, t-test and the Fisher’s exact test. The level of significance was set at 0.05.

## Results

The results showed that in all groups obturated with Adseal nor the type of irrigant neither the canal dryness condition, affected bond strength (*P*>0.05). The failure mode in this group was mostly cohesive.

In the AH-Plus samples although the difference in bond strength between the irrigants was not significant (*P*>0.05), the moisture condition strongly affected the bond strength (*P*˂0.05). Meaning that the bond under the dry condition was the strongest while in wet/half-wet condition, it was the lowest. In the half-wet and wet conditions no significant differences were observed (*P*>0.05). For AH-plus the failure mode was mostly mixed and cohesive in the NaOCl and CHX samples. In Endosequence groups, in any canal conditions there was no significant difference between the irrigants; although in the CHX samples, the push-out mean value was lower than that of NaOCl (*P*˃0.05). The failure mode for Endosequence in the CHX group under wet conditions was mostly cohesive, even under wet and dry conditions of NaOCl group. Under dry conditions of CHX the failure mode was mostly adhesive. Under half-wet condition of CHX the failure mode was mostly mixed. Under wet and half-wet conditions of NaOCl the failure mode was mostly cohesive (Tables 1 and 2).

**Table 1:** The mean (SD) of push-out test measured in Newtons in the study groups

**Sealer type**	**Irrigant **	**Canal condition**	**Push-out N **	***P*** **-value**
**Adseal**	NaOCl	wet	1.62 (0.55)	*P *˃0.05
		half-wet	1.61 (0.45)	
		dry	1.89 (0.65)	
	CHX	wet	1.74 (0.23)	
		half-wet	1.71 (0.70)	
		dry	1.64 (0.33)	
**AH-Plus**	NaOCl	wet	0.48 (0.27)	*P *˂0.05
		half-wet	0.20 (0.45)	
		dry	1.66 (0.08)	
	CHX	wet	0.48 (0.24)	
		half-wet	0.27 (0.43)	
		dry	1.60 (0.12)	
**Endosequence**	NaOCl	wet	0.71 (0.24)	*P *˃0.05
		half-wet	1.28 (0.63)	
		dry	1.22 (0.21)	
	CHX	wet	0.93 (0.31)	
		half-wet	0.82 (0.37)	
		dry	1.70 (0.54)	

**Table 2:** Distribution of failure modes in the study groups

**Sealer **	**Irrigant **	**Canal condition**	**Failure N (%)**		
			**Adhesive**	**Cohesive**	**Mixed**
**Adseal **	NaOCl	wet	1 (8.3)	8 (66.7)	3 (25.0)
		half-wet	2 (16.7)	8 (66.7)	2 (16.7)
		dry	2 (16.7)	6 (50.0)	4 (33.3)
	CHX	wet	1 (8.3)	4 (33.3)	7 (58.3)
		half-wet	0 (0.0)	9 (75.0)	3 (25.0)
		dry	1 (8.3)	7 (58.3)	4 (33.3)
**AH-Plus **	NaOCl	wet	3 (25.0)	2 (16.7)	7 (58.3)
		half-wet	2 (16.7)	4 (33.3)	6 (50.0)
		dry	4 (33.3)	3 (25.0)	5 (41.7)
	CHX	wet	2 (16.7)	7 (58.3)	3 (25.0)
		half-wet	2 (16.7)	8 (66.7)	2 (16.7)
		dry	0 (0.0)	9 (75.0)	3 (25.0)
**Endosequence**	NaOCl	wet	0 (0.0)	9 (75.0)	3 (25.0)
		half-wet	0 (0.0)	4 (33.3)	8 (66.7)
		dry	0 (0.0)	7 (58.3)	5 (41.7)
	CHX	wet	0 (0.0)	8 (66.7)	4 (33.3)
		half-wet	1 (8.3)	6 (50.0)	5 (41.7)
		dry	8 (66.7)	4 (33.3)	0 (0.0)

## Discussion

The aim of canal obturation is to seal the root canal system in order to prevent reinfection of the periapical tissues. The moisture and liquids can negatively influence the sealing ability of any given obturating material as they can inhibit or retard the setting reaction or even enhance it. These influences might increase leakage [18].

Likewise, bonding of sealer to dentin might be influenced by moisture conditions of the root canal system before obturation. Recent studies have shown that excessive dryness of the dentin can remove water remaining in the tubules and consequently endanger the penetration of hydrophobic sealer, affecting the quality of bonding [13]. It has been shown that different amounts of moisture remaining in the root canal can influence the sealing ability of resin-based sealers so that the bonding quality between the dentin and sealer is affected by the amount of moisture left in the root canal before obturation [19].

Various methods have been used for testing bond strength of root canal sealers such as shear bond strength, micro-tensile strength and push-out tests. The latter is reproducible and can be interpreted easily [20]. In this study we used push-out test to compare the bond strength of the sealers. Mohammadi [21] reported that NaOCl is the most common endodontic irrigant; however due to dentin degeneration and remaining amount of irrigant on the surface of the canal walls, oxygen production and interaction with bonded resin polymerization the bond strength might decrease.

Contrary to Morris* et al*. [22] who reported that high concentrations of sodium hypochlorite do not alter sealer-dentin interface, in the present study with resin-based sealers there was no significant difference between sodium hypochlorite and CHX. The lowest bond strength in AH-Plus group was under wet conditions, which is consistent with the results reported by Nagas *et al*. [19]. 

Our results showed that Adseal is less influenced by moisture although it is a resin-based sealer; this might be due to its shorter setting time compared to other sealers of the study. There are not sufficient studies available on this new sealer. Further studies should be done for more evaluation of this sealer. 

Under the wet condition there was no significant difference between the irrigants with Endosequence sealer but under dry condition the higher values of push-out test were seen in the NaOCl group and the difference was significant. As mentioned before, this sealer uses the moisture in the dentinal tubules for setting [19]. Shokuhinejad *et al*. [23] stated that the moisture in the dentinal tubules might not be enough for setting of this material but our study showed the highest values of bond strength of Endosequence in dry group. This results might not be a matter of comparison because Shokuhinejad *et al*. [23] used PBS moisture which is a different method compared to our study. The Endosequence BC sealer is mainly composed of calcium and silicate. Formation of apatite is a common phenomenon in calcium silicate-containing biomaterials [24, 25]. However, the excessive moisture in wet and half-wet groups resulted in a significant decrease in bond strength that might be because of confounding effect of excessive moisture on setting of this sealer. In our study the best result was achieved in the dry group that might be due to the remaining moisture in the canal even after drying with paper point, which is also stated by Nagas *et al*. [19], who found the highest bond strength of iRoot SP (a bioceramic sealer) in the moist group of their study.

In the present study, the most frequent failure mode with Adseal was observed with NaOCl irrigation solution under wet conditions which was cohesive in nature. This shows that the good adherence of the sealer to dentinal walls. In relation to CHX irrigation solution, the most frequent failure mode under wet conditions was of mixed type that shows adherence to both dentinal walls and within the sealer. However, cohesive failure under dry and half-wet conditions shows better adherence compared to wet groups. In relation to AH-Plus sealer, the most frequent failure modes in all three groups were mixed and cohesive with the use of NaOCl and CHX as irrigation solutions, respectively. This is similar to the study by Saleh *et al*. [26] who reported that the most frequent failure was cohesive with the use of AH-Plus sealer. 

Sealers like Adseal and BC sealer are rather newly introduced and many studies can be done on different aspects of their physical or even biological properties in future. 

## Conclusion

The bond strength of Adseal was not affected by the canal condition or irrigation type. Though AH-Plus was not affected by the irrigant type, the highest bond strength was seen in dry canals. For Endosequence BC sealer, the canal conditions did not affect the bond strength, however, CHX reduced the bond strength.
